# Extralysosomal cathepsin B in central nervous system: Mechanisms and therapeutic implications

**DOI:** 10.1111/bpa.13071

**Published:** 2022-04-12

**Authors:** Junjun Ni, Fei Lan, Yan Xu, Hiroshi Nakanishi, Xue Li

**Affiliations:** ^1^ Key Laboratory of Molecular Medicine and Biotherapy, Department of Biology, School of Life Science Beijing Institute of Technology Beijing China; ^2^ Department of Medical Genetics & Cell Biology, School of Basic Medical Sciences Zhengzhou University Zhengzhou China; ^3^ Department of Pharmacology, Faculty of Pharmacy Yasuda Women's University Hiroshima Japan; ^4^ Department of Stomatology, The Affiliated Hospital of Qingdao University Qingdao University Qingdao China; ^5^ School of Stomatology Qingdao University Qingdao China

**Keywords:** cathepsin B, central nervous system, leakage, lysosome, neurodegeneration, neuron development

## Abstract

Cathepsin B (CatB) is a typical cysteine lysosomal protease involved in a variety of physiologic and pathological processes. It is expressed in most cell types and is primarily localized within subcellular endosomal and lysosomal compartments. Emerging scientific evidence indicates that lysosomal leaked CatB is involved in mitochondrial stress, inflammasome activation, and nuclear senescence, but without the acidic environment. CatB is also secreted as a myokine, which is involved in muscle‐brain cross talk and neuronal dendritic remodeling. Lysosomal‐leaked and cellular‐secreted CatB functions are dependent on its enzymatic activity at a neutral pH. In the present review, we summarize the available experimental evidence that mechanistically links extralysosomal CatB to physiological and pathological functions in central nervous system, and their potential for use in therapeutic approaches.

## Graphical abstract

Emerging scientific evidence indicates that lysosomal leaked CatB is involved in mitochondrial stress, inflammasome activation, and nuclear senescence, but without the acidic environment. CatB is also secreted as a myokine, which is involved in muscle‐brain cross talk and neuronal dendritic remodeling. In the present review, we summarize the available experimental evidence that mechanistically links extralysosomal CatB to physiological and pathological functions in central nervous system, and their potential for use in therapeutic approaches

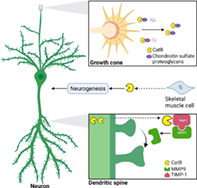

## 1 INTRODUCTION

Sixty‐five years ago, the lysosomes were discovered by Christian de Duve and his colleagues, who defined it as a monolayer vesicle that is rich in acidic hydrolases [1]. It is primarily known for degrading cellular macromolecules. However, in recent studies, they have emerged as crucial regulators of cell homeostasis, serving as a terminal destination during phagocytosis and autophagy and sensing cellular signals in coping with stress stimuli [2]. Lysosomes are monolayer membrane organelles surrounded by a 7‐10‐nm‐thick lipid membrane. The membrane vacuolar‐type H^+^‐ATPase (V‐ATPase) is a distinct characteristic of lysosomes and can continuously pump H^+^ into lysosomes to maintain an acid environment [3]. In addition to V‐ATPase, other membrane proteins, such as lysosome‐associated membrane proteins (LAMPs), ion channels, and multiple transporters, are the molecular bases for lysosomal function [4]. Under normal conditions, glycosylation of lysosomal membrane proteins effectively maintains membrane stability by preventing the lysosomal membrane from being destroyed by lumen proteolytic enzymes. However, loss of lysosomal membrane integrity, which is often referred to as lysosomal membrane permeabilization (LMP), occurs following persistent exposure to oxygen radicals, optical damage, and other factors, thereby resulting in the release of the internal enzymes into the cytoplasm [5, 6]. When LMP is not repaired, persistent lysosomal rupture may lead to the massive release of the lysosomal contents, and extensive cytoplasmic acidification, which results in irreversible cell damage [5].

Cathepsin B (CatB, EC 3.4.22.1), originally named CatB1, was the first and remains the best‐characterized member of the C1 family of papain‐like, lysosomal cysteine peptidases. First purified to homogeneity from the human liver in 1973 [7], it was found to be ubiquitously expressed in most cell and tissue types. Increasing evidence of the pathological roles and substrates of CatB has been reported as CatB knockout mice were established in 2000 [8]. In the last two decades, deregulated CatB synthesis and activity have been reported to be involved in the pathology of several diseases, such as cancer [9, 10, 11, 12], pancreatitis [13], liver fibrosis [14], rheumatoid arthritis [15, 16], inflammatory pain [17], traumatic brain injury [18], hypoxia‐ischemic brain injury [19], Alzheimer's disease [20, 21, 22, 23], and COVID‐19 infection [24, 25]. Although no CatB inhibitor has been approved for drug use, one has completed the Phase I trial for fatty liver disease [26], and another is in the late preclinical stage for treating Chagas disease [27].

## 2 CATHEPSIN B

### 2.1 Post‐translational processing and maturation of CatB

**FIGURE 1:**
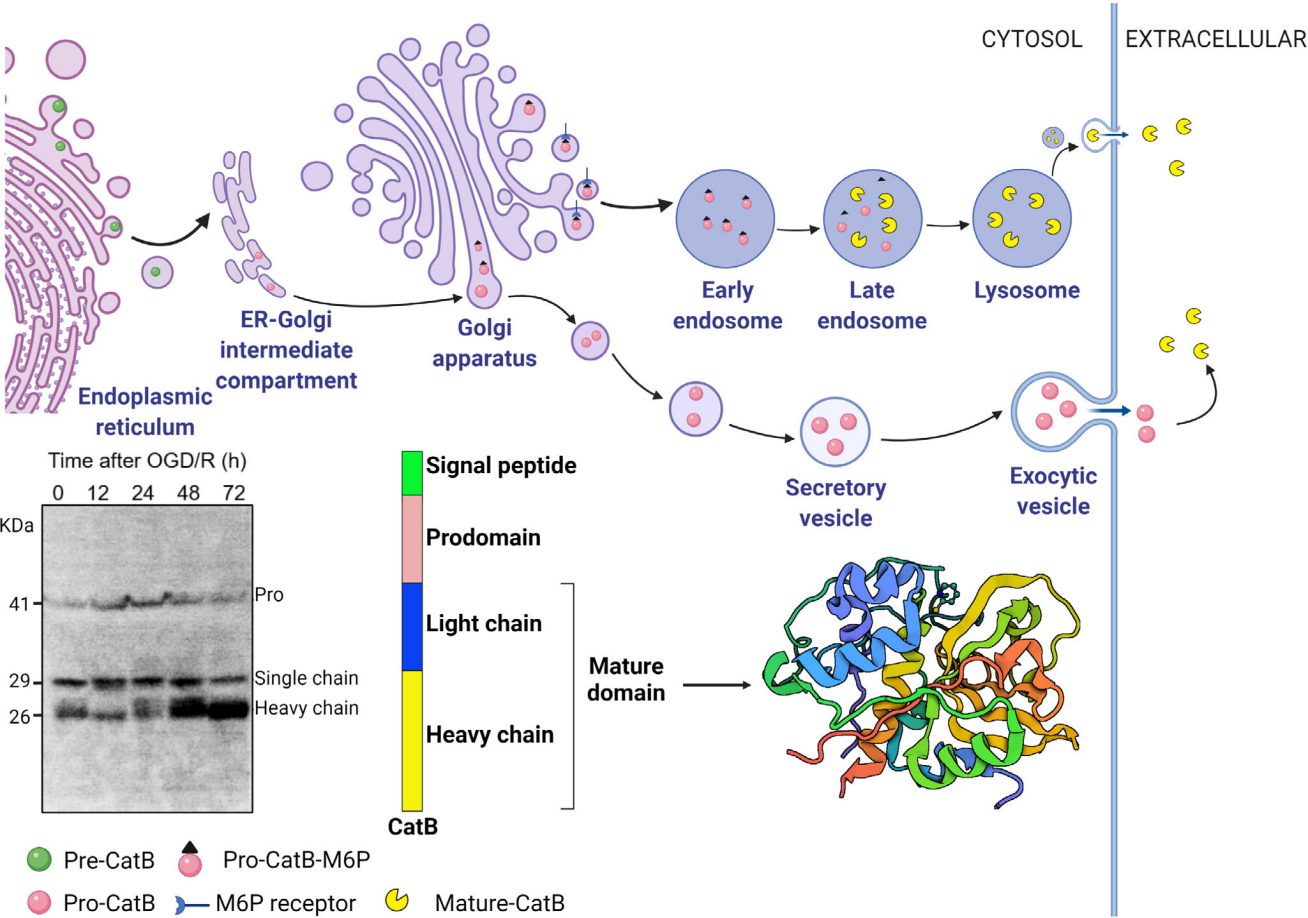
Schematic illustration of cathepsin B (CatB) proteolytic maturation. CatB is synthesized as a preform in the endoplasmic reticulum (ER). The N‐terminal signal peptide directs pre‐CatB into the lumen where they are cleaved, which generates pro‐CatB. Pro‐CatB later travels through the Golgi stocks, where the mannose residues are modified to mannose 6‐phosphate (M6P). The M6P‐tagged pro‐CatB are recognized and bound by M6P receptors in the trans‐Golgi network and are directly sorted into the endo/lysosomes. Pro‐CatB autoproteolytically activates itself into the mature form in acidic conditions. The left bottom panel is an immunoblotting image showing the maturation of CatB in microglia cells after oxygen–glucose deprivation and reoxygenation (OGD). The right bottom panel is the crystal structure of the tetragonal form of human liver CatB (Protein Data Bank ID: 2IPP)

CatB has a similar structure as that of other cysteine Cats and is composed of left (L)‐ and right (R)‐subdomains. Following synthesis as an inactive pre‐proenzyme by ribosomes associated with the endoplasmic reticulum (ER), pre‐CatB with an N‐terminal signal peptide targets this protein to the lumen of ER. After removal of the signal peptide (pre) from the ER lumen, pro‐CatB is delivered to and passed through the different stacks of Golgi apparatus, where pro‐CatB is modified in their N‐glycosidically linked oligosaccharide chains with mannose‐6 phosphate (M6P) residues. This unique post‐translational modification is critical for the correct intracellular targeting of pro‐CatB. M6P and M6P receptor mediated transport of pro‐CatB from the trans‐Golgi network to endo/lysosomes. Within the endo/lysosomes, the pH drops, and pro‐CatB is further processed via autocatalysis into a mature two‐chain form composed of an N‐terminal light chain and a C‐terminal heavy chain [28] (Figure 1). In mouse primary microglia and MG6 microglia cell lines, we detected the above forms of CatB; it is first synthesized as a 41‐kDa precursor, which is then converted into the mature, single‐chain intermediate enzyme (29 kDa) and further processed into the two‐chain enzyme (Figure 1, 26‐kDa heavy chain and 5‐kDa light chain).

In addition to the lysosome sorting pathway, CatB has been reported to enter the secretory pathway via the default mechanism because of insufficient M6P receptor‐mediated lysosomal sorting [29]. Under normal conditions, CatB occurs in the pericellular environment only as their latent precursors. However, enzymatically active extracellular forms of CatB have been found in tumors and plasma [30, 31]. These phenomena can be explained by the autoactivation of Pro‐CatB or the regulated exocytosis of mature‐CatB from lysosomes (Figure 1).

### 2.2 CatB expression in the brain

CatB has been reported to be involved in the pathology of a wide range of human diseases. In a complete genome analysis to search differential expressed genes in bipolar disorder (BD) and attention deficit hyperactivity disorder (ADHD) using RNA extracted from the peripheral blood of patients aged 7–23 years [32], CatB was found to be one of the 82 genes that were higher in ADHD than in BD. Although the study lacked healthy subjects as a control, the finding demonstrated the potential role of CatB in psychiatric disorders [32]. Furthermore, increased CatB expression and enzymatic activity were found to initiate neurovascular inflammation in autism spectrum disorder (ASD) [33]; however, the precise role of CatB in the pathology of ASD remains to be determined. Recently, we deleted the CatB gene using clustered regularly interspaced short palindromic repeats technology in differentiated neuron‐2a cells and found that these cells failed to produce neurite outgrowths, but the wild‐type (WT) did [34]. These findings provide valuable insight into the physiological function of CatB in neural development.

In addition to the role of CatB in developmental pathology, increasing evidence has demonstrated its pathological role in adulthood and aged individuals. Notwithstanding the large amount of research conducted on the roles of CatB in the pathological conditions, its proteolytic function can be divided into the following categories: (1) trigger inflammation by activating the inflammatory switch in myeloid cells; (2) degrade the extracellular matrix (ECM), thereby facilitating the invasive potential of tumor cells; (3) promote the production of vital pathological molecules by activating specific substrates.

## 3 MECHANISMS OF LEAKED CatB‐MEDIATED CELL STRESS AND DEATH

Although CatB exhibits broad and redundant substrate specificities, it can carry out highly specialized functions. This is achieved through strict regulation of its expression and activity in distinct subcellular compartments. Importantly, CatB works as a carboxypeptidase in the endolysosomal system, although CatB also has endopeptidase activity at a neutral pH [28]. It has been reported that CatB retains its proteolytic functions in the cytoplasm, nucleus, or extracellular environment despite suboptimal pH conditions.

### 3.1 Cytosol‐located CatB

**FIGURE 2:**
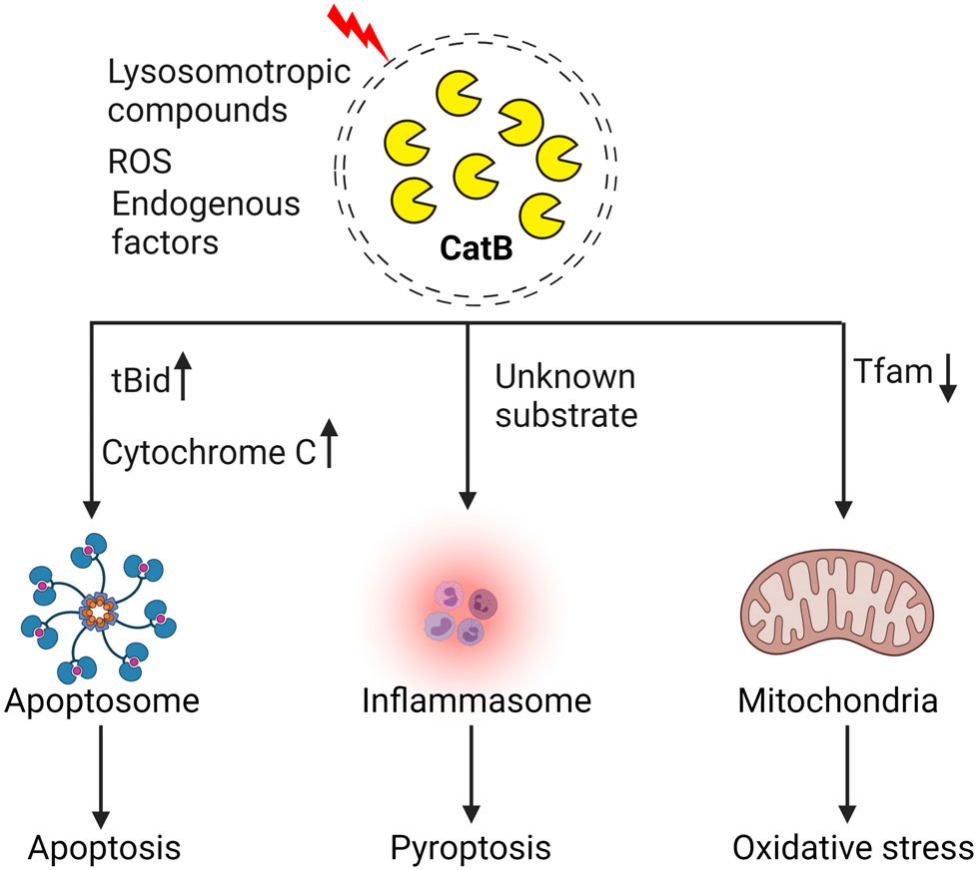
Pathological function of cytosol located CatB. CatB translocates from the lysosome to the cytosol when the cell is challenged with lysosomotropic compounds, reactive oxidative species (ROS), or endogenous factors. Cytosol CatB induces apoptosis, pyroptosis, and oxidative stress, through the cleavage of specific substrates including Bid and Tfam

The most studied leaked CatB is the cytosol translocated CatB, because LMP can be induced by a plethora of stimuli, such as lysosomotropic compounds with detergent activity, reactive oxygen species (ROS), and several endogenous cell death factors (Figure 2). In contrast to the massive lysosomal breakdowns that induce cytosolic acidification and subsequent cell death, we discuss the partial and selective LMP, which persist as the primary lysosomal function and maintain a cytosolic neutral environment. Although extensive research has been conducted on cytosol‐located CatB (Table 1), the molecular mechanisms underlying small‐scale lysosomal leakage and translocation of CatB to cytosol remain unclear. Recently, however, Yuan et al. [50] have hypothesized that the inactivation of *N*‐ethylmaleimide sensitive factor (NSF) ATPase, which regulates the membrane trafficking pathway from the Golgi apparatus to the endolysosomal compartments, is responsible for small‐scale release of CatB in neurons after brain ischemia. CatB can be further released on a large scale from substantially more damaged endolysosomal compartments into the cytoplasm and eventually into the extracellular space.

**TABLE 1:** Experimental evidence for extralysosomal CatB‐activated substrate in diseases pathology

Expression in specific cells/tissues	CatB localization	Experimental method used to determine localization	Substrates	Pathology	References
Acinar cell	Cytosol	Cytosolic fraction with western blotting and enzymatic activity	Trypsin	Pancreatitis	Saluja et al. [35]; Talukdar et al. [36]
Neuron	Cytosol	Immunoelectron microscopy and immunostaining	N. D	Traumatic brain injury	Lafrenaye et al. [37]
Liver	Cytosol	Enzymatic activity assay	CASP‐11	Apoptosis, Inflammation	Schotte et al. [38]
Liver	Cytosol	Cytosolic extracts with enzymatic activity assay	Bid	Apoptosis	Schotte et al. [38]
Microglia, Human PBMCs	Cytosol	Co‐staining CatB with LAMP	N. D	AD, NLRP3 activation	Halle et al. [39]; Hornung et al. [40]
Myeloid‐derived suppressor cells	Cytosol	Co‐IP CatB with NLRP3	LRR domain of NLRP3	NLRP3 activation, Cancer	Bruchard et al. [41]
Rat hepatoma cells (McA‐RH7777)	Cytosol	Immunostaining of CatB, Cytosolic fraction with western blotting	LFABP	Hepatic steatosis	Thibeaux et al. [42]
Microglia	Cytosol	Enzymatic activity assay	Pre‐Tfam	Aging	Ni et al. [43]
HeLa cells	Mitochondria	Co‐staining CatB with MitoTracker	N. D	Osteoarthritis	Muntener et al. [44]
Microglia	Nucleus	Enzymatic activity assay and nuclear fraction of Western blotting	Sirtuins	Aging	Meng et al. [45]
Thyroid carcinoma cells	Nucleus	Immunostaining	Histone H1	Thyroid malignancies	Tedelind et al. [46]
Human pancreatic cancer cells, HT22 cells	Nucleus	Immunostaining	Histone H3	Ferroptosis	Kuang et al. [47]; Nagakannan et al. [48]
Type 1 alveolar epithelial cells	Exosomes	Mass spectrometry	N. D	Oxidative stress	Downs et al. [49]
Rat hepatoma cells (McA‐RH7777)	Golgi apparatus	Immunostaining of CatB	LFABP	Hepatic steatosis	Thibeaux et al. [42]

#### 3.1.1 Cytosol CatB in apoptosis

Vandenabeele's group first reported that CatB can efficiently process pro‐caspases 1 and 11 but only weakly process the precursors of caspases 2, 3, 6, 7, and 14 at both acidic and neutral pH levels [38, 51]. Another example of the implication of the link between lysosomal CatB and caspase and cell death is that activated caspase 8 induces the release of CatB from lysosomes. Cytosol CatB triggers the release of cytochrome c through cytosolic Bid and subsequent apoptosis, which is downstream of the Bid cleavage [52]. In experimental pancreatitis, active trypsin within the co‐localized organelles makes these organelles fragile and leaky, through which CatB leaks out into the cytosol where the cytosol CatB induces Bid cleavage and cytochrome c release from mitochondria, leading to apoptosis of acinar cells [37]. Notably, CatB has also been reported to limit necroptosis through the cleavage of receptor‐interacting protein kinase‐1 in macrophages [53]. The anti‐necroptotic effects of CatB fit well into the paradigm of pro‐apoptotic factors acting to inhibit necroptosis.

#### 3.1.2 Cytosol CatB in pyroptosis

Additionally, cytosol CatB is capable of executing cell death, entirely independent of the apoptotic machinery. One such example is pyroptosis, which is a form of inflammatory programmed cell death [54]. The first report on the involvement of CatB in the innate immune response was by Halle et al. [39], who demonstrated that cytosol CatB acts “upstream” of the assembly of nucleotide‐binding oligomerization (NOD)‐, leucine‐rich repeat (LRR)‐, and pyrin domain (PYD)‐containing protein 3 (NLRP3) inflammasomes using pharmacological inhibition of CatB by CA074Me. Furthermore, similar results were demonstrated in human peripheral blood mononuclear cells in the presence of silica crystals [40]. However, whether cytosol CatB interacts directly with the inflammasome or whether this process involves intermediary molecules activated by CatB was not explored in the above studies. It was further shown that CatB directly interacts with NLRP3 using co‐precipitation and surface plasmon resonance assay in vitro [41]. To confirm the interactive domain of NLRP3 with CatB, Bruchard et al. [41] generated recombinant NLRP3 protein with deletion of the PYD, the NOD domain or the LRR domain. They found that CatB interacted with NLRP3 with deletion of the PYD or NOD fragment but not with NLRP3 with deletion of the LRR fragment.

The issue about the involvement of cytosol translocated‐CatB in the NLRP3 activation seemed to be settled. However, this issue is still a matter of argument, because the involvement of other cysteine cathepsins cannot be ruled out [55, 56]. In mammalian cells, only small amounts of a cell‐permeable CA‐074Me are converted to CA074, a specific inhibitor of CatB, resulting in additional inhibition of cathepsins [57]. Moreover, a variety of cathepsins, including CatB, X, L, or S, exhibit the function of promoting NLRP3 priming and activation, which is also blocked by CA‐074Me [58]. In addition, a potential NLRP3‐independent role for autolysosomal CatB in the procaspase‐1 activation and the subsequent interleukin‐1β secretion has been identified [59, 60]. Additional studies are necessary to elucidate the precise roles of CatB in the procaspase‐1 activation.

#### 3.1.3 Cytosol CatB in aging

In aged microglial cells, we found that cytosol‐translocated CatB is responsible for the degradation of mitochondrial transcription factor A (Tfam), which is closely associated with the stabilization of mitochondrial DNA (mtDNA) structure. The increased generation of mitochondria‐derived ROS and proinflammatory mediators through impaired mtDNA biosynthesis in microglia during aging [43]. We also provided evidence that the lateral ventricular injection of CatB‐overexpressed microglia treated with L‐leucyl‐L‐leucine methyl ester, a lysosome‐destabilizing agent, was sufficient to induce cognitive impairment in middle‐aged mice. Injection of CatB‐overexpressed microglia without treatment of L‐leucyl‐L‐leucine methyl ester failed to induce cognitive impairment, suggesting that cytosol‐translocated CatB drives chronic neuroinflammation and brain aging.

#### 3.1.4 Physiological roles of cytosol CatB

**FIGURE 3:**
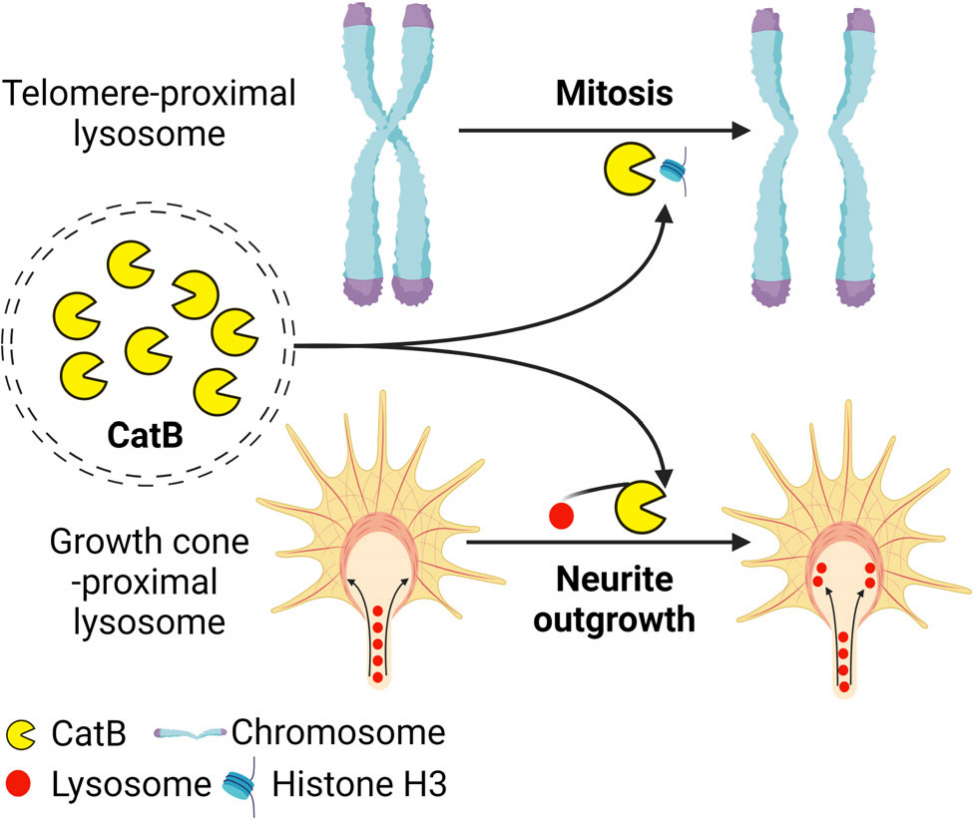
Physiological function of cytosol located CatB. Telomere‐proximal lysosomes leak CatB into the cytosol, where CatB promotes the segregation of telomeres during mitosis through the cleavage of histone H3. Cytosol CatB from growth cone‐proximal lysosomes may be involved in lysosome trafficking and remodeling during neurite outgrowth

Notwithstanding the large amount of work carried out on the pathological roles of cytosol CatB, lysosomal‐leaked CatB also played a physiological role in the maintenance of genomic integrity. CatB from telomere‐proximal lysosomes during cell mitosis mediates the cleavage of a subset of histone H3 (Figure 3), which contributes to the appropriate segregation of telomeres in cultured human cells and healthy murine tissues [61]. In primary cultured neurons and neuro‐2a cells, we found that CatB deletion significantly inhibits neurite outgrowth. The punctate staining signals of CatB and LAMP2 were well co‐localized and located at the neuritic edge of neurons (Figure 3), which suggests that CatB is involved in the initiation of neurite outgrowth [34]. We proposed that cytosol CatB may work as “pacesetters” at the leading edge of the growth cone; however, the molecular mechanism through which CatB intracellularly regulates the initiation of neurite outgrowth remains to be elucidated. In addition, cathepsin D, another typical cathepsin, was shown to be involved in the maintenance of lamellipodia extension as a cytosolic translocated form [62].

### 3.2 Mitochondrial‐located CatB

The CatB gene comprises 12 exons, and its message consists of several variants produced by alternative exon splicing [63, 64]. The splice variant lacking exons 2 and 3 has been detected initially as a pare species in human breast and colon carcinomas and human melanoma [64]. The above splice variant encodes a protein that lacks the signal peptide and propeptide, which was demonstrated to be delivered to the mitochondria instead of the lysosomes [44]. Moreover, cells expressing such a variant died following nuclear fragmentation, which suggested apoptosis [65, 66]. However, CatB has been shown to lyse mitochondrial membranes and promote cytochrome c release into the cytosol, which subsequently results in cell death [67, 68]. Although the spice variant and lysosome‐leaked CatB have been examined in mitochondria, the transportation and exact location of CatB in mitochondria remain unclear. Mitochondria contain two major membranes and are the critical location for ATP synthesis; therefore, studies on the mechanism of lysosome‐mitochondria CatB translocation and its subsequent pathological functions are of value.

### 3.3 Nuclear‐located CatB

The nucleus is the largest organelle in mammalian cells and provides a site for genetic transcription. Cats in the nucleus processes transcription factors that control cell cycle progression, which facilitates cell proliferation and differentiation [69].

In aged microglia, the enzymatic activity of CatB is significantly increased after LMP, and we revealed that the nucleus‐translocated CatB was involved in the cleavage of sirtuins (Sirts), which were discovered as longevity factors because of their role in age‐associated metabolic disorders and stress resistance [45]. Therefore, the inhibition of nuclear CatB or blockage of its nuclear translocation may prevent cellular senescence during aging. Interestingly, depletion of Sirt1 in invasive breast cancer cells increases the secretion of non‐vesicular CatB. When co‐administered with exosomes from Sirt1‐deficient cells, CatB induces invasive behavior of MCF10AT1 cells in vitro [70, 71]. The above evidence collectively suggests that nuclear CatB is involved in the degradation of Sirt1, which results in further secretion of CatB into the extracellular space. Cellular self‐protection via the balancing of nuclear and extracellular CatB cannot be ruled out.

Moreover, nuclear CatB has been reported to digest histone H1 in thyroid carcinoma cells, which demonstrates its role in the development of thyroid malignancies [46]. In principle, nuclear CatB can cleave a large variety of nuclear substrates because of its broad substrate specificity. However, results from in vitro digestion assays have indicated that histone H1 is specific for CatB‐mediated cleavage. Other studies have also reported nuclear CatB‐mediated nuclear damage during the process of ferroptosis, which is a type of non‐apoptotic regulated cell death involving excessive iron accumulation and subsequent lipid peroxidation [47]. Soon after, using pharmacological and genetic models of ferroptosis, the substrate of nuclear CatB in ferroptotic cell death was shown to be histone H3 [48].

### 3.4 Other compartments located CatB

#### 3.4.1 Exosomal CatB

Exosomes are nano‐sized vesicles that are secreted into the extracellular environment. These vesicles contain various biological effector molecules that regulate intracellular signaling pathways in recipient cells. It has been reported that exosomal CatB regulates the expression of receptors for advanced glycation end‐product in Type 1 alveolar cells under conditions of oxidative stress [49]. However, the Melendez group demonstrated that macrophage‐derived exosomes are a transport machinery to deliver CatB from macrophage to neurons in the presence of human immunodeficiency virus, which leads to neuronal dysfunction [72]. Consequently, exosomal CatB may provide a common use or compensation between different cell types. In our studies, it has not been possible to induce cell‐specific deletion of Cats, even in conditional knockout mice because the target cells endocytose pro‐Cat secreted by neighboring cells through M6P‐ specific receptors on the cell surface. After endocytosis, pro‐Cat can be delivered to lysosomes, where it is converted into a mature form. Indeed, CatD can still be observed in neurons in neuron‐specific CatD‐deficient mice, which are generated by mating CatD‐floxed mice with nestin‐Cre mice. Therefore, further studies using CatB conditional knockout mice should consider the intercellular transportation of CatB.

#### 3.4.2 Golgi apparatus located CatB

As discussed in Section 2.1, pro‐CatB are modified in N‐glycosidically linked oligosaccharide chains with M6P residues in the Golgi apparatus. Few studies have focused on pro‐CatB in the Golgi apparatus. In a lipid homeostasis study, Thibeaux et al. [42] found that CatB was co‐localized with liver fatty acid‐binding protein (LFABP) in the cytosol as well as in the Golgi apparatus. However, the exact role and functional type of CatB in LFABP in the apparatus was not elucidated. It has been reported that NLRP3 inflammasome activation occurs in the dispersed trans‐Golgi network (dTGN), which serves as a scaffold for NLRP3 aggregation in the signaling cascade [73]. CatB has been widely accepted as the critical factor for NLRP3 activation, despite the lack of evidence. Considering the presence of CatB in dTGN, the possible role of CatB from dTGN or on dTGN in the activation of NLRP3 should be considered.

### 3.5 Extracellular‐located CatB

#### 3.5.1 Physiological roles of extracellular CatB

The presence of CatB in extracellular space can be achieved via the secretory pathway and lysosomal exocytosis [29, 74, 75, 76]. Although extracellular CatB is more commonly observed during pathological conditions, its physiological functions have also been recently identified (Table 2).

**TABLE 2:** Experimental evidence for the extracellular CatB‐activated substrate

Expression in specific cells/tissues	CatB localization	Experimental method used to determine localization	Substrates	Physiologies/pathologies	References
Neuron	Extracellular	Inhibition of CatB by CA‐074	MMP‐9	Long term structural plasticity of dendritic spines	Padamsey et al. [77]
Neuron	Extracellular	Western blotting of DRG CM	Chondroitin sulfate proteoglycans	Axon outgrowth	Tran et al. [78]
Skeletal muscle cells	Extracellular	Mass spectrometry	N. D	Hippocampal neurogenesis	Moon et al. [31]
Astrocytoma and glioblastoma	Extracellular	Immunohistochemistry	Tenascin‐C	Tumor angiogenesis	Mai et al. [79]
Thyroid carcinoma	Extracellular	CatB activity examination	Type I and IV collagen	Extra‐capsular invasion and lymph node metastasis	Kusunoki et al. [80]
Rat chondrosarcoma chondrocytes	Extracellular	In vitro assay	Proteoglycan and collagen	Arthritis	Maciewicz and Wotton [81]

**FIGURE 4:**
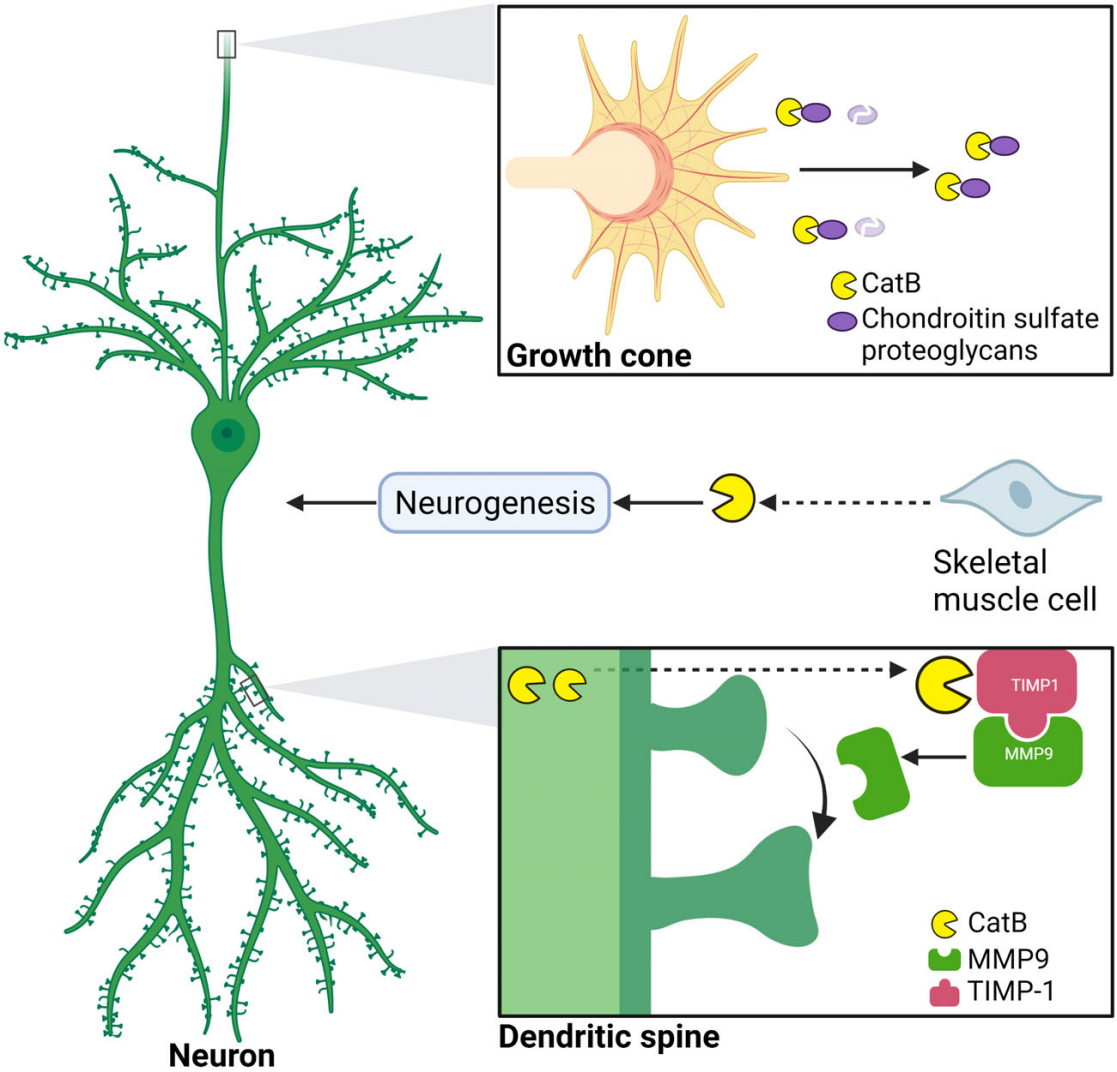
Extracellular CatB is involved in neuronal plasticity and regeneration. Extracellular CatB is involved in neurite outgrowth through the degradation of chondroitin sulfate proteoglycans, dendritic spine plasticity through the degradation of tissue inhibitor of metalloproteinase (TIMP) 1, and in neurogenesis

Both lysosomes and the plasma membrane contain negatively charged lipids in their outer and inner layers, respectively. Therefore, calcium ions (Ca^2+^) could bridge the opposing charges on the membrane. In addition, lysosomes have been suggested to function as a Ca^2+^ store. Padamsey et al. [77] found that lysosomal Ca^2+^ signaling regulates exocytosis of CatB from lysosomes, which regulates the long‐term structural plasticity of dendritic spines by triggering matrix metalloproteinase 9, an enzyme involved in extracellular matrix remodeling and synaptic plasticity. The extracellular CatB has also been reported to enhance axonal outgrowth in neurons through the degradation of chondroitin sulfate proteoglycans, a major axon inhibitory matrix [78]. In addition to the direct effects on the brain, exercise has been shown to regulate brain function through secretion of skeletal muscle‐derived factors, called myokines, and CatB has been identified as a myokine that enhances adult hippocampal neurogenesis and spatial memory function in response to exercise [31]. These findings demonstrate the central role of extracellular CatB in neuronal synaptic plasticity and cognitive function (Figure 4).

#### 3.5.2 Pathological roles of extracellular CatB

In addition to the intracellular roles of CatB, secreted CatB has also been implicated in a variety of diseases involving tissue remodeling states, such as Alzheimer's disease and tumor metastasis via the degradation of extracellular matrix components [82].

Gan et al. [83] have conducted functional genomic approaches to identify CatB as one of the genes transcriptionally induced by amyloid β peptide 42 (Aβ_42_) in microglia. They have further shown that CA‐074, an irreversible CatB‐specific inhibitor with very low membrane permeability, completely abolished the toxicity in the conditioned medium released by activated microglia [83]. These observations suggest that extracellular CatB released from Aβ_42_‐activated microglia is a critical mediator in causing neuronal death, probably through degradation of extracellular matrix compartments. CatB is also secreted by tumor cells as a pre‐form, whereas many types of tumor cells release the mature, active form of CatB [84, 85]. When CatB knockout mice were crossed with a transgenic cancer model, reduction in tumor initiation, proliferation, angiogenesis, and invasion were reported [86]. Moreover, Mai et al. [79] reported the degradation of the extracellular matrix protein, tenascin‐C, by CatB, which is involved in the progression of gliomas. Thyroid carcinomas with extra‐capsular invasion and metastasis were found to have high CatB for type I and IV collagen degrading abilities [80]. Furthermore, extracellular CatB has been found to degrade both proteoglycan and collagen components, as seen in arthritis [81]. In summary, extracellular CatB has a high ability to degrade the matrix and offers dual functions in different situations. It promotes neuronal plasticity during neuronal development and cancer progression in various cancers. Thus, regulators that control the dual functions of CatB and specific intervention windows may become particularly important.

## 4 REMAINING QUESTIONS ON EXTRALYSOSOMAL CatB

### 4.1 Regulation of LMP

Massive lysosomal breakdown may induce cytosolic acidification, which in turn can cause cell death; hence, the term “suicide bags.” Partial and selective LMP may be involved in the leakage of CatB, which may lead to various pathologies. Despite extensive characterization of leaked CatB in cell stress and death, the molecular mechanical link between LMP and CatB remain unclear. For instance, how is LMP regulated and controlled endogenously? Can CatB be precisely regulated to leak? Is CatB involved in LMP?

Several reports have indicated that not all lysosomes are permeabilized simultaneously. Giant lysosomes appear to be particularly susceptible to the action of LMP‐inducing reagents [87]. In addition, the production of intracellular mediators such as ROS, which have a spatially limited range of activity, may induce LMP in lysosomes in only those subcellular regions near mitochondria, which are the major ROS‐generating organelles [88]. Following this principle, lysosomes that are localized in the proximity of uncoupled mitochondria are more likely to suffer damage to their membranes than are distant lysosomes. Interestingly, using high spatial and temporal resolution confocal live‐cell imaging, mitochondria and lysosomes were found to make contact, allowing bidirectional regulation of mitochondrial and lysosomal dynamics, which may explain the dysfunction observed in both organelles in various human diseases [89]. The above research also demonstrated that lysosomes functionally regulate mitochondrial fission through direct contact [89], and new mitochondrial DNA synthesis enables NLRP3 inflammasome activation [90]. CatB has been reported to modulate lysosomal biogenesis in defense against *Francisella novicida* infection [91]. Taken together, these findings point to CatB in lysosome‐mitochondria communications and the pathogenesis of multiple diseases being linked to mitochondrial and lysosomal dysfunction.

### 4.2 Transportation of CatB

Extracellular CatB is secreted from vesicles or exocytosed from the lysosome; however, the molecular mechanism underlying the translocation of lysosomal‐leaked CatB into other organelles remains unclear. Gong et al. [64] reported that a CatB splice variant lacking exons 2 and 3 encodes a protein that lacks the signal peptide and the pro‐peptide, which is delivered to the mitochondria instead of the lysosome. Mitochondria contain an outer membrane and inner membrane, with a small intermembrane space in between. Approximately 99% of mammalian mitochondrial proteins are encoded by the nuclear genome and are synthesized as precursors in the cytosol and imported into the mitochondria by mitochondrial protein import machinery [92]. The imported protein is critically controlled by import machinery, which includes the translocase of the outer membrane complex, translocase of the inter membrane (TIM)22 and TIM23 complexes, mitochondrial import and assembly complex, and the sorting and assembly machinery and mitochondrial import machinery complexes [92]. We previously found mitochondrial‐located CatB in microglia cells in the presence of L‐leucyl‐L‐leucine methyl ester. Whether the types of import machinery are involved in the translocation of CatB into mitochondria needs to be ascertained. It is worth noting that the lysosome‐destabilizing agent may also impair the integrity of the mitochondrial membrane, which may result in the diffusion of cytosol CatB into mitochondria.

### 4.3 Reliable strategies to detect extralysosomal CatB

The commonly used method to examine extralysosomal CatB is to separate and detected indicated fractions using western blotting. However, the lysosome membrane can also be impaired during the fraction separation procedure, which may produce artificial results. We aimed to mark the extralysosomal CatB through immunofluorescent staining using its monoclonal antibody; however, we were unable to detect a positive signal in microglia cells by treatment with a lysosome‐destabilizing agent. Nevertheless, we detected extralysosomal CatB enzymatic activity when Z‐Arg‐Arg‐cresyl violet was applied, which is a cell‐permeable fluorescently labeled CatB substrate, the fluorescent cresyl violet group of which is designed to be unquenched upon cleavage of one or both of the arginines by CatB [45, 93]. Therefore, the enzymatic activity assay should be more sensitive than immune labeling in terms of leaked CatB detection.

CA‐074Me, a membrane‐permeable selective inhibitor of CatB, and E64d, a broad cysteine protease inhibitor, are used most frequently for pharmacological inhibition of CatB. However, it is noted that the effects of CA‐074Me and E64d are pH‐dependent. They are strong inhibitors for CatB at pH 4.5 in the lysosomal luminal acidic environment, while ineffective inhibitors for released CatB at the cytosolic neutral pH [94]. Recently, Hook's group found that CatB possesses pH‐dependent cleavage preferences that can be utilized for the design of a selective neutral pH inhibitor, and Z‐Arg‐Lys‐AOMK was designed and revealed as a potent and selective neutral pH inhibitor of CatB [95]. The neutral pH inhibitor has more than 100‐fold greater potency at pH 7.2 compared with that at pH 4.6, which provides a valuable opportunity to study the role of pathogenic functions of CatB at neutral pH in human diseases.

## 5 PERSPECTIVES ON LEAKED AND EXTRACELLULAR CatB

### 5.1 Real‐time examination of CatB

In situ real‐time fluorescence imaging is a useful method to observe the dynamics of enzymes and their interactions with other species in the biosystem. CatB expression and leakage usually occur at specific stages during the progression of cell stress and death in human diseases, the time window in which CatB leak is critical for pathological and pharmacological studies. Studies have suggested that CatB expression or activity peaks during early‐stage cancer and subsequently declines with advanced disease [96, 97]. Therefore, developing a new method with high binding efficiency toward CatB for in situ real‐time imaging enzymes remains a significant challenge and opportunity. Furthermore, a CatB‐specific bioluminescence probe, Val‐Cit‐AL, was designed for selectively sensing CatB activity in vitro with a 67‐folds “turn on” of bioluminescence intensity, and is also capable of sensing CatB activity in living cells and tumors [98]. Applying the luciferase‐luciferin bioluminescence system for CatB sensing to the pathological progression of various diseases will be valuable and interesting in future studies.

### 5.2 CatB as biomarkers

CatB is expressed in most cell types and becomes upregulated in a variety of diseases, as mentioned in Section 2.1. High levels of CatB are readily measurable in tissues, lipid biopsies, and cerebral spinal fluid using standard, colorimetric assays. For instance, enzyme‐linked immunosorbent assay (ELISA) analysis on sera obtained from patients with nasopharyngeal carcinoma showed that the CatB concentration was 12.5 ± 3.5 mg/L; in healthy controls, it was 2.5 ± 1.4 mg/L [99]. Although changes in CatB content may occur as a consequence of disease or injury, a method with increased accuracy, markedly reduced background noise, and the need of only limited biological sample volumes may enable the detection of small changes in CatB levels in blood samples in the early stages of human diseases. Thangavelu et al. [100] developed a digital ELISA for differential detection of CatB with less than 5 μl of serum and plasma using a single molecular array (SiMoA) platform, which offers 1000 times more sensitivity and vastly reduced variance compared with those of colorimetric tests. The lower limit of quantitation of CatB was approximately 2.3 pg/ml in the buffer and approximately 9.4 pg/ml in the serum or plasma, which demonstrated the ability of SiMoA to measure small changes above endogenous levels in blood samples [100]. Because of the improved detection ranges and differential quantitation from the baseline measurement, this customized assay has utility in both basic research and clinical settings.

### 5.3 Cell type‐specific delivery of the CatB inhibitor

Different cell types belong to distinct lineages and are developmentally specified through integrated transcriptional and epigenetic control of cell differentiation and gene expression [101]. In terms of CatB, the expression and function in the brain are different, and a series of studies have suggested its pro‐inflammatory role in microglia in brain diseases, which include hypoxic ischemia, inflammatory pain, aging, and Alzheimer's disease (AD) [17, 19, 43, 45, 102]. However, neuronal CatB has been conversely reported to function in amyloid β degradation and production in AD [21, 22, 23]. Therefore, studies on cell type‐specific delivery of CatB inhibitors will improve the precision of CatB function and provide more evidence for precision medicine.

Upon intravascular administration, therapeutics encounters several intravascular barriers, which include intravascular enzymes that degrade the active ingredients, clearance of molecules of a certain size (<6 nm) via renal filtration, clearance of nanoparticles by phagocytes [103]. The other extremely restrictive barrier is the endothelial barrier between the blood and the brain (blood–brain barrier [BBB]) [104]. Upon entering the brain, therapeutics with intracellular targets have to precisely recognize the target cells and cross the plasma membrane to function in the cytosol or other cell organelles. Therefore, the development of a new CatB inhibitor must avoid intravascular degradation, phagocyte clearance, the BBB, cell‐specific targeting, and cell membrane permeability.

## 6 CONCLUSION

This review of extralysosomal CatB highlights their essential roles in the development and progression of various diseases and physiological processes, and discusses the regulatory mechanisms and clinical implications. It may be possible to prevent and treat related disorders caused by lysosomal CatB leakage by inhibiting CatB in the extralysosomal space without affecting its classical degrading function in lysosomes. The development of a cell‐type specific inhibitor of CatB with only neutral pH flexibility that has minimal or no side effects is being continued, while facing considerable challenges.

## 7 OUTSTANDING QUESTIONS

The translocation of CatB is a dynamic process and its roles in neuronal function are complex. While existing data suggest significant promise for CatB intervention in reducing and treating inflammatory neuronal disorders, a great deal of work remains to identify disease‐stage‐specific therapy.

1. To this aim better elucidation of the extralysosomal CatB function is required to identify key master regulators of the complex signaling network underlying brain pathology.
2. The study of the differential modification of CatB in physiological and pathological conditions seems promising for disease‐specific inhibitor development.
3. Further development of administration methods and formulations is required to ensure efficient brain delivery of CatB inhibitor at therapeutically meaningful concentrations.
4. Development of in vivo extralysosomal probe for real‐time imaging will provide much needed for diagnostic methods and early warning of patients with brain disorders.

## 8 SEARCH STRATEGY AND SELECTION CRITERIA

Data for this review were identified by search of PubMed. Google Scholar and references from relevant articles using the search terms “cathepsin B,” “neuron,” “brain,” “neurodegenerative disease,” “neuron development,” and “lysosome permeability.” Only articles published in English between 1956 and 2021 were included. The final reference list was generated on the basis of relevance and originality with regard to the topics covered in this review.

## CONFLICT OF INTERESTS

The authors declare no conflict of interests.

## AUTHOR CONTRIBUTIONS

Junjun Ni: conceptualization; funding acquisition; writing—original draft; writing—review & editing; visualization. Fei Lan, Yan Xu, Hiroshi Nakanishi: writing—review & editing; visualization. Xue Li: conceptualization; funding acquisition; writing—review & editing; visualization. All authors read and approved the final version of the manuscript.

## Data Availability

Data sharing is not applicable to this article as no new data were created or analyzed in this study.
